# ABC-GOALScl score predicts admission to the intensive care unit and mortality of COVID-19 patients over 60 years of age

**DOI:** 10.1186/s12877-023-03864-8

**Published:** 2023-03-10

**Authors:** María Elena Camacho-Moll, Zayra Ramírez-Daher, Brenda Leticia Escobedo-Guajardo, Julio César Dávila-Valero, Brenda Ludmila Rodríguez-de la Garza, Mario Bermúdez de León

**Affiliations:** 1 Department of Molecular Biology, Center for Biomedical Research at Northeast, Mexican Social Security Institute, Calle 2 de abril 501, Col. Independencia, Monterrey, Nuevo Leon 64720 Mexico; 2 Center for Molecular Diagnosis and Personalized Medicine, Health Sciences Division, Universidad de Monterrey, Av. Ignacio Morones Prieto 4500-Pte, Zona Valle Poniente, Nuevo Leon 66238 San Pedro Garza Garcia, Mexico; 3 Residency in Geriatrics, Zone General Hospital No. 4, Mexican Social Security Institute, C. Mariano Matamoros 300, Centro de Guadalupe, Guadalupe, Nuevo Leon 67100 Mexico

**Keywords:** Admitting department, Hospital, Aged, Critical care, Geriatrics, Hospitalized, Pandemics

## Abstract

**Background:**

One of the risk factors for getting seriously ill from COVID-19 and reaching high mortality rates is older age. Older age is also associated with comorbidities, which are risk factors for severe COVID-19 infection. Among the tools that have been evaluated to predict intensive care unit (ICU) admission and mortality is ABC-GOALScl.

**Aim:**

In the present study we validated the utility of ABC-GOALScl to predict in-hospital mortality in subjects over 60 years of age who were positive for SARS-CoV-2 virus at the moment of admission with the purpose of optimizing sanitary resources and offering personalized treatment for these patients.

**Methods:**

This was an observational, descriptive, transversal, non-interventional and retrospective study of subjects (≥ 60 years of age), hospitalized due to COVID-19 infection at a general hospital in northeastern Mexico. A logistical regression model was used for data analysis.

**Results:**

Two hundred forty-three subjects were included in the study, whom 145 (59.7%) passed away, while 98 (40.3%) were discharged. Average age was 71, and 57.6% were male. The prediction model ABC-GOALScl included sex, body mass index, Charlson comorbidity index, dyspnea, arterial pressure, respiratory frequency, SpFi coefficient (Saturation of oxygen/Fraction of inspired oxygen ratio), serum levels of glucose, albumin, and lactate dehydrogenase; all were measured at the moment of admission. The area under the curve for the scale with respect to the variable of discharge due to death was 0.73 (IC 95% = 0.662—0.792).

**Conclusion:**

The ABC-GOALScl scale to predict ICU admission in COVID-19 patients is also useful to predict in-hospital death in COVID-19 patients  ≥ 60 years old.

## Background

In December 2019, the people’s Republic of China reported rare cases of pneumonia of unknown origin. The first cases were traced back to a seafood market called “Huanan” in Wuhan, Hubei province. Clinical symptoms included fever, dyspnea, and bilateral lung infiltrates [1]. In January 2020, the identification of the coronavirus responsible for this disease was described. This was a Betacoronavirus RNA, which was named Severe Acute Respiratory Syndrome Coronavirus-2 (SARS-CoV-2) [2]. By March 11, 2020, this disease was declared a pandemic by the World Health Organization, and to date, there have been more than 5 million cumulative deaths worldwide, with a global lethality of 1.9% [3]. Among the risk factors for infection and developing severe COVID-19, age, sex, comorbidities, obesity, and tobacco smoking have been included [4–8]. In Mexico, to date there are more than 300,000 cumulative deaths, and the prevalence of COVID-19 in older patients represents 19% [3]. According to global reports, most infected people’s age is around 50 years; however, there is increased mortality in people over 60 years old [9]. The average age of fatal cases is 80 years based on Italian reports, where only 1.1% of deaths occurred in people younger than 50 years old [10]. The association of age as a risk factor has also been published elsewhere [11].

Scales to predict possible outcomes are suggested tools for treatment decisions; for instance, the National Institute for Health and Care Excellence guidelines are evidence-based recommendations for health and care in England that assess frailty through a clinical frailty score. This score takes into consideration comorbidity, function, and cognition and classifies patients from 1 (very healthy) to 9 (terminally ill). Those whose score is less than 5 are eligible for complete and invasive support in the intensive care unit (ICU) [12]. ABC-GOALSc is another promising tool that has been demonstrated to be useful to predict ICU admission in COVID-19 patients, with an area under the curve of 0.79 and 0.77 in the development and validation cohorts, respectively. This result has been improved by adding other factors to the model, where ABC-GOALScl had an area under the curve of 0.86 and 0.87 in the development and validation cohorts, respectively, and ABC-GOALSclx had an area under the curve of 0.88 and 0.86 in the development and validation cohorts, respectively [13]. Differences between ABC-GOALScl and ABC-GOALSclx scores are that the latter includes a tomographic image analysis of thorax through the CO-RADS categories. The present paper aims to establish whether ABC-GOALScl is also useful to predict in-hospital mortality in COVID-19 patients over 60 years of age.

## Methods

The study was conducted according to the guidelines of the Declaration of Helsinki and approved by the Institutional Review Board of the Mexican Social Security Institute (protocol 2021–1909-106, August 9, 2021). This is a retrospective and non-interventional study, where medical records were consulted and data in the public repository are de-identified.

This study was carried out with records from patients hospitalized in the Zone General Hospital No. 4 “Villa Guadalupe” located in Guadalupe, Nuevo Leon, Mexico. Patients 60 of years old and older with confirmed COVID-19 diagnosis by RT-PCR test and hospitalized between December 1, 2020 and January 5, 2021 were included. A database was collected that included social security number, age, comorbidities, days of hospitalization, outcome, and date of discharge or death. Database, raw and processed are available at Mendeley Data, V1, https://doi.org/10.17632/z4z22nbmmz.1. ABC-GOALScl, which incorporates clinical and laboratory results, was used and scored as previously described [13]. This model includes sex, systolic arterial pressure (SAP), presence or absence of dyspnea by respiratory frequency (RF), Charlson comorbidity index, glucose serum levels, obesity, albumin serum levels, lactate dehydrogenase (LDH) serum levels, and SpFi coefficient (Saturation of oxygen/fraction of inspired oxygen, SO_2_/FiO_2,_ ratio). Subjects who had incomplete clinical records, were diagnosed with *Acinetobacter spp.* infection or *Clostridium difficile,* had records that came from another unit, or were directly admitted to the ICU were excluded. Files from subjects who voluntarily requested to leave the study were deleted.

The distribution of continuous variables was evaluated with Kolmogorov–Smirnov. Descriptive statistics were used to analyze the data; qualitative variables are described in frequencies and percentages. For comparison of qualitative variables, chi-squares and stepwise multivariate logistic ordinal regression models were run to calculate adjusted odds ratio (OR) and 95% Confidence Interval (CI) for each component of the ABC-GOALScl score. For quantitative data, a t–test and a Mann–Whitney U test were performed. Sensitivity, specificity, positive predictive value (PPV), negative predictive value (NPV), and the area under the curve (AUC) were calculated, and a value of *p* < 0.05 was considered significant.

## Results

A total of 243 subjects over 60 years of age diagnosed with COVID-19 were included in this study. The average age was 71.7, and males represented 57.6%. Average days of hospitalization were 11.53. At least one comorbidity was reported in 206 patients (84.8%). The Charlson comorbidity index had an average score of 4, which would fall into the moderate category. Average Body Mass Index (BMI) was 28.7. Average systolic arterial pressure was 127 mm Hg, average O_2_ saturation was 80%, average FiO_2_ was 40%, and the average SpFi coefficient (SO_2_/FiO_2_ ratio) was 294.8. Measured laboratory variables were glucose, albumin, and LDH. The average glucose value was 190.8 mg/dL, 3.2 g/dL for albumin, and 411 U/L for LDH (Table 1). The outcome of 145 subjects (59.7%) was death, whereas 98 recovered (40.3%). Finally, the average ABC-GOALScl result was 8.2 (Table 1). Table 2 summarizes the contributions of factors in the final ABC-GOALS score. Mainly, age, total comorbidities, BMI, respiratory frequency, and SO_2_/FiO_2_ ratio were the most determinant factors (Table 2).

**Table 1:** Characteristics of subjects (*N* = 243) included in the study

	**N (%)**
Sex (N)	
Male	140 (57.6)
Female	103 (42.4)
Comorbidities (N)	
Any	206 (84.8)
Hypertension	181 (74.5)
Diabetes mellitus	129 (53.1)
Chronic kidney disease	73 (15.2)
COPD	15 (6.2)
Oncological disease	10 (4.1)
Dementia	6 (2.5)
Autoimmune disease	3 (1.2)
Liver disease	2 (0.8)
Age (years)	
60–65	59 (24.3)
66–70	59 (24.3)
71–75	55 (22.6)
≥ 76	70 (28.8)
Charlson Comorbidity Index (N)	
Mild 1–2	50 (20.6)
Moderate 3–4	113 (46.5)
Severe ≥ 5	80 (32.9)
Outcome (N)	
Death	145 (59.7)
Recovery	98 (40.3)
ABC-GOALScl (N)	
0–3 Low risk	12 (4.9)
5–9 Moderate risk	148 (60.9)
≥ 10 High risk	83 (34.2)
	**Mean (Min, Max)**
Days of hospitalization	11.53 (1, 110)
Total comorbidities (N)	1.6 (0, 4)
BMI	28.7 (16.7, 59.03)
Systolic arterial pressure (mmHg)	130.7 (52, 220)
Respiratory frequency (breaths/min)	25.5 (12, 48)
O_2_ saturation (sO_2_)	0.8 (0.15, 0.99)
fraction of inspired oxygen (FiO_2_)	0.4 (0.21, 1.00)
sO_2_/FiO_2_ ratio	294.8 (18.75, 466.67)
Laboratory results	
Glucose (mg/dL)	190.8 (32, 1097)
Albumin (g/dL)	3.2 (1.38, 4.34)
LDH (U/L)	411 (110, 2950)

*COPD* chronic obstructive pulmonary disease, *BMI* body mass index, *sO*_*2*_ saturation of oxygen, *FiO*_*2*_ fraction of inspired oxygen, *LDH* lactate dehydrogenase

**Table 2:** ABC-GOALScl components

	**Death** **N (%)**	**Recovery** **N (%)**	**Chi- square** ***p*-value**	**OR (Min, Max)**	***p*-value**
Sex (N)					
Men	86 (59.3)	54 (55.1)	0.52	1	
Women	59 (40.7)	44 (44.9)		0.94 ( 0.50, 1.78)	0.852
Age (years)					
60–65	51 (35.2)	19 (19.4)	0.052	1	
66–70	32 (22.1)	23 (23.4)		0.92 (0.40, 2.10)	0.843
71–76	30 (20.7)	29 (29.6)		1.15 (0.63, 3.57)	0.362
> 76	32 (54.2)	27 (27.6)		**3.19 (1.32, 7.68)**	**0.01**
Total comorbidities (N)					
0	13 (9.0)	24 (24.5)	0.021	1	
1	47 (32.4)	26 (26.5)		**3.35 (1.31, 8.53)**	**0.011**
2	62 (42.8)	33 (33.7)		**3.37 (1.32, 8.65)**	**0.011**
3	20 (13.8)	14 (14.3)		2.19 (0.72, 6.68)	0.169
4	3 (2.1)	1 (1.0)		2.02 (0.14, 29.20)	0.605
BMI					
< 29.99	101 (69.7)	79 (80.6)	0.056	1	
> 30	44 (30.3)	19 (19.4)		**2.39 (1.13, 5.05)**	**0.023**
Dyspnoea (N)					
No	7 (4.8)	4 (4.1)	0.78	1	
Yes	138 (95.2)	94 (95.9)		0.89 (0.21, 3.87)	0.876
SAP (mm Hg)					
> 101	128 (88.3)	88 (89.8)	0.71	1	
< 100.9	17 (10.2)	10 (10.2)		1.33 (0.41, 4.37)	0.635
RF (breaths/min)					
< 23	55 (37.9)	45 (45.9)	0.001	1	
24–28	54 (37.2)	47 (48)		0.79 (0.42, 1.47)	0.456
> 29	36 (24.8)	6 (6.1)		**4.30 (1.48, 12.46)**	**0.007**
Glucose (mg/dL)					
< 199	92 (63.9)	78 (79.6)	0.009	1	
> 200.9	53 (36.6)	20 (20.4)		1.68 (0.83, 3.37)	0.148
Albumin (g/dL)					
> 3.5	42 (29.0)	40 (40.8)	0.055	1	
< 3.49	103 (71.0)	58 (59.2)		1.40 (0.74, 2.67)	0.306
LDH (U/L)					
< 199.9	14 (9.7)	16 (16.3)	0.12	1	
> 200	131 (90.3)	82 (83.7)		1.88 (0.79, 4.44)	0.152
sO_2_/FiO_2_ ratio					
> 300	65 (44.8)	69 (70.4)	0.000	1	
< 299.9	80 (55.2)	29 (29.6)		**2.32 (1.25, 4.29)**	**0.008**

*BMI* Body mass index, *OR* Odds ratio, *SAP* Systolic Arterial Pressure, *RF* Respiratory Frequency, *LDH* Lactate Dehydrogenase, *sO*_*2*_*/FiO*_*2*_ Saturation of Oxygen/Fraction of Inspired Oxygen

A significant relationship between subject outcome and ABC-GOALScl score was observed by the chi-square test. Logistic regression analysis also demonstrated that the ABC-GOALScl score is a useful tool to predict subject outcomes, where subjects classified with moderate risk in the ABC-GOALScl classification had 5 times the probability of death, and subjects classified with high risk had 24.6 times higher probability (Table 3).

**Table 3:** ABC-GOALScl *chi*-square and multivariate linear regression against outcome

**ABC-GOALScl**	**Death** **N (%)**	**Recovery** **N (%)**	**Chi-square** ***p*-value**	**OR (Min, Max)**
Low risk	2 (1.4)	10 (10.2)	0.000	1
Moderate risk	74 (51.0)	74 (75.5)		5.00 (1.06, 23.60)*
High risk	69 (47.6)	14 (14.3)		24.64 (4.86, 124.93)***

^*^, *p* < 0.05; ***, *p* < 0.001. *OR* Odds ratio

**Fig. 1:**
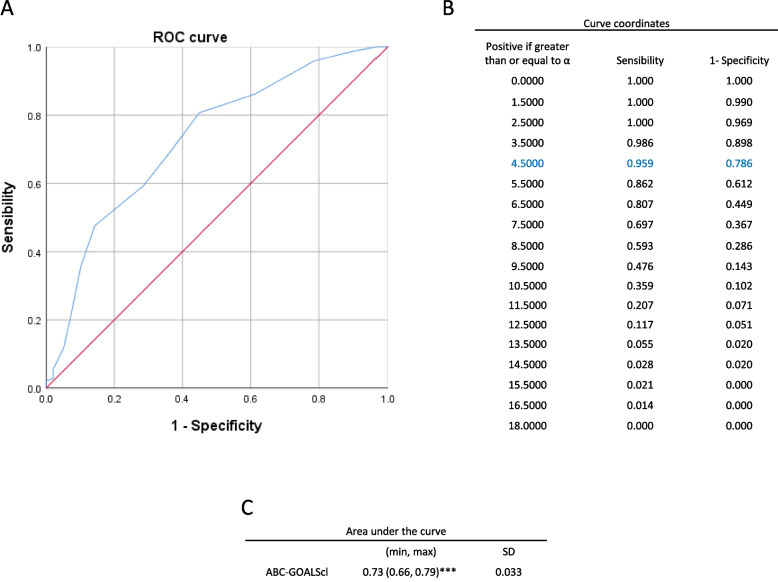
Estimating the risk of death using ABC-GOALScl. **A** Receiver operating characteristic (ROC) curve; **B** curve coordinates; **C** Area under the curve results for ABC-GOALScl. ***, *p* < 0.001. SD, standard deviation

The ABC-GOALScl model demonstrated good accuracy in estimating the risk of death, with an area under the curve of 0.7, with 0.96 sensitivity and 0.79 for 1–specificity values at 4.5 as the best cutoff point (Fig. 1). PPV and NPV were also calculated, resulting in 83.1% and 52.5%, respectively, when grouping low– with moderate–risk compared to high-risk patients based on their ABC-GOALScl score.

## Discussion

The present study aimed to establish whether the ABC-GOALScl score is useful to predict in-hospital death in a subject cohort over 60 years of age infected with COVID-19. It is well known that advanced age is a risk factor for COVID-19 infection and mortality. Aging is also a trigger for comorbidity development, which at the same time reduces the probability of a good outcome after COVID-19 infection. Multiple tools such as the ABC-GOALScl score have been validated to predict ICU admission and mortality due to COVID-19 in the general population. We have found a 73% probability that ABC-GOALScl will predict subject outcome.

There are other scores specifically developed for in-hospital mortality. They have been developed by using parameters to evaluate respiratory function, oxygen saturation, and some markers of inflammatory processes, all common events in pulmonary diseases, among other variables that support the original intended goal of the score. Examples include the Clinical Characterization Consortium (ISARIC‐4C) score with an area under the ROC curve (AUROC) of 0.799 (0.738 – 0.851); the COVID‐GRAM Critical Illness Risk Score (COVID‐GRAM), with AUROC of 0.785 (0.723 – 0.838); the quick COVID‐19 Severity Index (qCSI), with an AUROC of 0.749 (0.685 – 0.806); and the National Early Warning Score (NEWS), with an AUROC of 0.764 (0.700 – 0.819) [14]. Adding to this, the present study reports that the ABC-GOALScl score has an AUROC of 0.73 (0.66 – 0.79), which is very similar to the results of other scores (Table 4); however, the sensitivity and specificity balance was better for ABC-GOALScl with our data, even when inflammatory parameters such as interleukin 6 and neutrophil-to-lymphocyte ratio were not considered. Median age, male prevalence, Charlson comorbidity index, and sample size make our results similar to those reported by Covino and others (2020) [14].

**Table 4:** Scoring systems for predicting death of COVID‐19 patients over 60 years of age

**Score**	**AUROC (Min, Max)**	**N**	**Cut off value**	**Sensitivity (%)**	**Specificity (%)**	**PPV**	**NPV**
NEWS	0.764 (0.700, 0.819)	210	> 4	66.7	69	35	49.2
COVID‐GRAM	0.785 (0.723, 0.838)	210	> 17.7	88.1	61.3	36.3	95.4
ISARIC‐4C	0.799 (0.738, 0.851)	210	> 8	88.1	5.9	33.3	94.9
qCSI	0.749 (0.685,0.806)	210	> 5	69	77.4	43.3	90.9
PSI (65–84)	0.85 (0.80, 0.90)	438	91	100	38.3	23.9	98.5
PSI (> 85)	0.69 (0.60, 0.79)	201	91	100	19.8	14.9	94.7
CURB-65 (65–84)	0.73 (0.65, 0.82)	438	N/A	65.5	74.7	16.3	96.5
CURB-65 (> 85)	0.60 (0.48, 0.73)	201	N/A	47.4	65.9	12.7	92.3
**ABC-GOALScl**	**0.73 (0.66, 0.79)**	**243**	**4.5**	**95**	**78.6**	**83.1**	**52.5**

*AUROC* Area Under the ROC Curve, *NEWS* National Early Warning Score, *COVID-GRAM* COVID-GRAM Critical Illness Risk Score, *ISARIC-4C* International Severe Acute Respiratory Infection Consortium Clinical Characterization Protocol-Coronavirus Clinical Characterization Consortium, *qCSI* quick COVID-19 Severity Index, *PSI* Pneumonia Severity Index, *CURB-65* confusion, blood urea, respiratory rate, blood pressure, pneumonia Severity Score [14, 15]

Glucose levels are a significant predictor of mortality in our study. None of the other scores included this parameter. Considering that more than 10% of people in Mexico suffers from diabetes mellitus and it is the third cause of death after COVID-19 [16], our score better fits the characteristics of the Mexican population. Nonetheless, the associated number of comorbidities constitutes an important predictor of mortality in ISARIC-4C and COVID-GRAM scores, as in our study [17–19].

The average BMI of 28.7 represents a population with overweight characteristics. In our study, this was not a predictor of mortality, but Bartoletti et al. (2020) reported in a similar score (PREDI-CO) that obesity is a stronger condition for the outcome in hospitalized patients with COVID-19. In geriatric people, risk of malnutrition is also a common feature. In contrast with our results, evidence of a relationship between malnutrition and mortality has been reported using the CONUT score. This event could be explained by the differences among populations [20, 21].

García-Gordillo et al. (2021) compared a newly developed score named COVID-IRS against ABC-GOALScl and six other scores to predict the risk of invasive mechanical ventilation in infected patients with COVID-19. ABC-GOALScl had a performance with an AUC intermediate to the newly developed and other implemented scores. Respiratory failure represents the principal cause of death in hospitalized COVID-19 patients [22].

Other groups have reported models for in-hospital mortality in the general population. A Chinese group has also reported a model that includes age, history of hypertension, and coronary heart disease, with an area under the curve of 0.88 (95% CI 0.80 – 0.95), sensitivity of 92.31%, and specificity of 77.44%; however, this model is for the general population, not for geriatric patients [23]. This same laboratory developed a model to predict in-hospital mortality, but based on age and lab results (high-sensitivity C-reactive protein, peripheral capillary oxygen saturation, neutrophil and lymphocyte count, d-dimer, aspartate aminotransferase, and glomerular filtration), with an AUC of 0.83 (0.68–0.93) [23].

Other studies have described sex, increased fraction of inspired oxygen, and crackles as the best predictors of mortality, with 4, 1, and 2.4 times increased probability of mortality, respectively [24]. ABC-GOALScl includes these factors and agrees with Mendes and colleagues’ results (2020).

Mesas and colleagues (2020) published a systematic review that included 60 studies, in which they investigate predictors of in-hospital mortality by gender, age, and health parameters; they concluded that dyspnea is an important factor as well as obesity and several other comorbidities [25]. However, for geriatric subjects, the most important factors were obesity, albumin, total bilirubin, alanine aminotransferase, serum ferritin, C-reactive protein and LDH. The results published by these authors agrees with our study given that they reported similar factors to the ones included in the ABC-GOALScl score. Differences can be attributed to the size and design of the different studies.

We report a higher mortality rate in subjects over 60 years of age (59.7%) compared to other studies, where it has been reported to be around 32% [25, 26]; this could be explained by the fact that our hospital was designated to concentrate patients with severe COVID-19 disease. Another explanation could be that the patient’ records were considered as completed in a longer period of time compared to other studies, where records included for the study were those completed in a month [18].

This study used a tool that has been widely validated to classify patients at risk of ICU admission and therefore at a higher risk of death [13]. A significant advantage of the ABC-GOALScl is that it allows follow-up of patients within the hospital, where the service for patients over 60 years of age can be personalized.

Due to the retrospective design of the present study, we can list some limitations. For instance, previous severe clinical conditions and treatments were not considered. The mortality rates may not be representative of the Mexican population because data were obtained from a hospital designated as a COVID-19 center during the pandemic period. We suggest performing more prospective studies to validate this model and to identify the key predictors for mortality in the population over 60 years of age.

## Conclusions

We conclude that ABC-GOALScl is a useful tool that could be applied in hospitals to give personalized treatments and interventions that might increase the favorable outcomes for patients over 60 years of age.

## Data Availability

Raw and processed data are available at Mendeley Data, V1, https://doi.org/10.17632/z4z22nbmmz.1 (https://data.mendeley.com/datasets/z4z22nbmmz/1).

## Abbreviations

AUC: Area Under the Curve
AUROC: Area Under the ROC Curve
BMI: Body Mass Index
CI: Confidence Interval
COPD: Chronic Obstructive Pulmonary Disease
COVID-19: Coronavirus Disease of 2019
FiO_2_: Fraction of Inspired Oxygen
ICU: Intensive Care Unit
LDH: Lactate Dehydrogenase
NEWS: National Early Warning Score
NPV: Negative Predictive Value
PPV: Positive Predictive Value
RF: Respiratory Frequency
RNA: Ribonucleic Acid
ROC: Receiver Operating Characteristic
SAP: Systolic Arterial Pressure
SO_2_: Saturation of Oxygen
SpFi: Saturation of Oxygen/Fraction of Inspired Oxygen coefficient
